# LLM-supported self-diagnosis and self-treatment of familial Mediterranean fever: a case report

**DOI:** 10.1016/j.ero.2026.100244

**Published:** 2026-09-29

**Authors:** Jessica Pauz, Phillip Kremer, Tingting Xiong, Martin Krusche

**Affiliations:** Department of Medicine III, University Medical Center Hamburg-Eppendorf, Martinistr, Hamburg, Germany

## Abstract

Large language models (LLMs) are increasingly being used by patients to seek explanations for persistent or unexplained symptoms. However, their impact on real-world diagnostic pathways and patient behavior remains poorly understood.

We report the case of a 34-year-old male of Egyptian origin with recurrent episodes of fever, severe migratory abdominal pain, arthralgia, myalgia, and intermittent erythematous skin lesions since early childhood. Despite almost 3 decades of extensive investigations, no unifying diagnosis had been established. During an iterative dialogue with the LLM DeepSeek in June 2025, the differential diagnosis was progressively refined toward familial Mediterranean fever (FMF). Although the model repeatedly recommended specialist assessment, it also provided specific information regarding genetic testing and colchicine treatment. The patient subsequently arranged genetic testing independently and obtained colchicine in Egypt, initiating treatment without prior specialist assessment at 0.5 mg twice daily. His inflammatory attacks ceased completely. However, liver enzymes increased, prompting temporary treatment discontinuation and dose reduction. FMF was thereafter confirmed by specialist assessment based on the characteristic clinical phenotype, family history, genotype, and response to colchicine. This case illustrates how patient-facing LLMs may facilitate recognition of rare autoinflammatory diseases.

However, confident diagnostic language combined with actionable treatment advice may also encourage unsupervised testing and self-medication, underscoring the need for clear safety boundaries and specialist confirmation.

A 34-year-old man born in Egypt presented to our autoinflammatory disease clinic in February 2026 after independently undergoing commercially available genetic testing suggestive of familial Mediterranean fever (FMF).

Since early childhood, he had experienced recurrent inflammatory attacks characterized by fever, severe migratory abdominal pain, arthralgia involving the knees, feet and hips, lower-limb myalgia, and intermittent erythematous skin lesions.

Episodes lasted ∼4 days, resolved between attacks, and gradually increased in frequency from 1 to 2 episodes annually during childhood to every 2 to 3 weeks in adulthood. During symptomatic episodes, C-reactive protein repeatedly exceeded 100 mg/L, reaching a documented maximum of 200 mg/L.

Relevant comorbidities included relapsing-remitting multiple sclerosis, which had remained stable for 7 years with fingolimod therapy. Family history was notable for recurrent unexplained abdominal pain in the patient’s father and recurrent febrile episodes in a cousin.

Despite almost 3 decades of symptoms, extensive investigations, including contrast-enhanced abdominal CT and MRI, gastroscopy, colonoscopy, and urological assessment, failed to establish a diagnosis. According to the patient, his primary care physician primarily arranged repeated laboratory testing. In addition, he was under regular neurological care for multiple sclerosis and had undergone urological evaluation for a urachal cyst, suspected neurogenic bladder dysfunction, recurrent urinary tract infections, and prostatitis. Overall, the previous diagnostic work-up had largely focused on gastrointestinal and urological causes without providing a unifying explanation for the recurrent episodes of fever and abdominal pain.

After extensive but inconclusive investigations, the patient began a dialogue with the publicly available LLM (DeepSeek-R1-0528) on June 28, 2025. The interaction was iterative and patient-led, with additional clinical information being disclosed over successive turns (full conversation, see Supplementary 1). Our reporting follows the Chatbot Assessment Reporting Tool (CHART) statement [1] as far as applicable to a single-patient report (Supplementary 2). In its initial response, the model proposed a broad differential diagnosis dominated by urological, infectious, and gastrointestinal explanations while also including FMF among several hereditary periodic fever syndromes. It recommended specialist evaluation, measurement of inflammatory markers during attacks, and consideration of genetic testing.

After the patient-reported normal contrast-enhanced abdominal imaging, normal bladder emptying, unrevealing gastroscopy and colonoscopy, and C-reactive protein concentrations consistently exceeding 100 mg/L during symptomatic episodes, the model deprioritized urological and neurological causes and increasingly favored an autoinflammatory disorder. When the patient later disclosed that attacks resolved completely after ∼4 days, FMF was presented as the leading diagnosis, with tumor necrosis factor alpha receptor–associated periodic syndrome and other periodic fever syndromes as alternatives.

Although the model continued to recommend rheumatological assessment and *MEFV* testing, its language became increasingly confident, at times describing the presentation as classic or highly suggestive of FMF. It also repeatedly discussed a diagnostic trial of colchicine and provided specific dosing suggestions before specialist assessment. In its final response concerning concomitant fingolimod treatment, however, it explicitly advised that colchicine should only be initiated with specialist approval and appropriate laboratory monitoring.

Motivated by the LLM-generated diagnostic hypothesis, the patient independently obtained commercial genetic MEFV testing at Alfa Labs, Egypt, which identified 2 pathogenic *MEFV* variants (c.2090A>G, p.Met694Val, and c.2177T>C, p.Val726Ala). Before specialist assessment, he obtained colchicine himself from a pharmacy in Egypt and initiated treatment at a dose of 0.5 mg twice daily without prior specialist supervision. Notably, although the LLM discussed colchicine treatment in detail and reviewed potential interactions with fingolimod, it repeatedly advised that treatment should only be initiated under specialist supervision. Following colchicine initiation, recurrent febrile attacks ceased completely for the first time since childhood. However, alanine aminotransferase increased to 160 U/L.

In view of the elevated liver enzymes, colchicine was temporarily discontinued, and liver enzyme levels were monitored closely by the primary care physician. After improvement in liver enzyme levels, colchicine treatment was resumed at a dose of 0.5 mg/d under close supervision. At presentation to our center, physical examination showed no evidence of active systemic inflammation while receiving colchicine 0.5 mg/d. Based on the characteristic lifelong clinical phenotype, Mediterranean ancestry, suggestive family history, identification of 2 pathogenic MEFV variants, and clinical response to colchicine, the diagnosis of FMF was confirmed [2]. The patient has remained free of attacks with regular clinical and laboratory follow-up.

This case illustrates several emerging aspects of patient-facing LLMs. Notably, the model did not simply produce a correct diagnosis from a static symptom list but continuously refined its differential diagnosis in response to newly disclosed clinical information. Throughout the conversation, alternative explanations including recurrent urinary tract disease, inflammatory bowel disease, and neurological complications were gradually discarded while FMF became increasingly likely. This conversational format may offer advantages over static symptom checkers [[3], [4]], although this cannot be inferred from a single case.

Equally important, however, are the limitations illustrated by this case. As the dialogue progressed, the LLM expressed increasing diagnostic confidence despite the absence of confirmatory investigations and continued to recommend several investigations that would probably not have been prioritised by an autoinflammation specialist once the characteristic phenotype had emerged [5]. Across 2 recent comparative studies involving FMF cases, LLMs showed lower diagnostic accuracy than physicians (30%-65% vs 70%-90%) [6,7], suggesting that LLMs may support diagnostic hypothesis generation but currently remain less accurate than clinicians.

More importantly, although the model consistently recommended specialist evaluation before treatment initiation, the patient nevertheless translated the generated diagnostic hypothesis into autonomous genetic testing and self-medication. The subsequent elevation of liver enzymes illustrates that even clinically accurate diagnostic suggestions may inadvertently encourage harmful behavior if patients perceive the model as sufficiently authoritative. The case suggests that generic recommendations to seek specialist advice may not sufficiently mitigate risk when they are accompanied by confident diagnostic language and actionable treatment instructions [8,9].

Furthermore, results from self-arranged genetic testing require expert interpretation. The detection of MEFV variants alone is not sufficient to diagnose FMF; variant pathogenicity, analytical validity of the assay, and concordance with the clinical phenotype must be considered. Direct-access results should therefore be clinically reviewed and, where appropriate, confirmed in an accredited diagnostic laboratory before treatment decisions are made.

Rather than replacing physicians, patient-facing LLMs may therefore be best understood as diagnostic navigators that facilitate recognition of uncommon disease patterns and shorten diagnostic delay [3]. This is particularly relevant in low-prevalence settings, where prolonged diagnostic delay and extensive diagnostic or surgical procedures remain common among patients with FMF [10]. German registry data additionally indicate long intervals between the onset of autoinflammatory attacks and the initiation of targeted treatment, highlighting persistent barriers to timely recognition and disease control [11].

The greatest value of LLMs may lie not in establishing diagnoses but in generating appropriate hypotheses and directing patients toward specialist evaluation (Fig). So far, currently available evidence does not support their use as autonomous diagnostic tools for FMF. Future studies should therefore move beyond benchmark diagnostic accuracy and investigate how LLM-assisted reasoning influences real-world patient behavior, diagnostic pathways, and patient safety [12].

**Figure:**
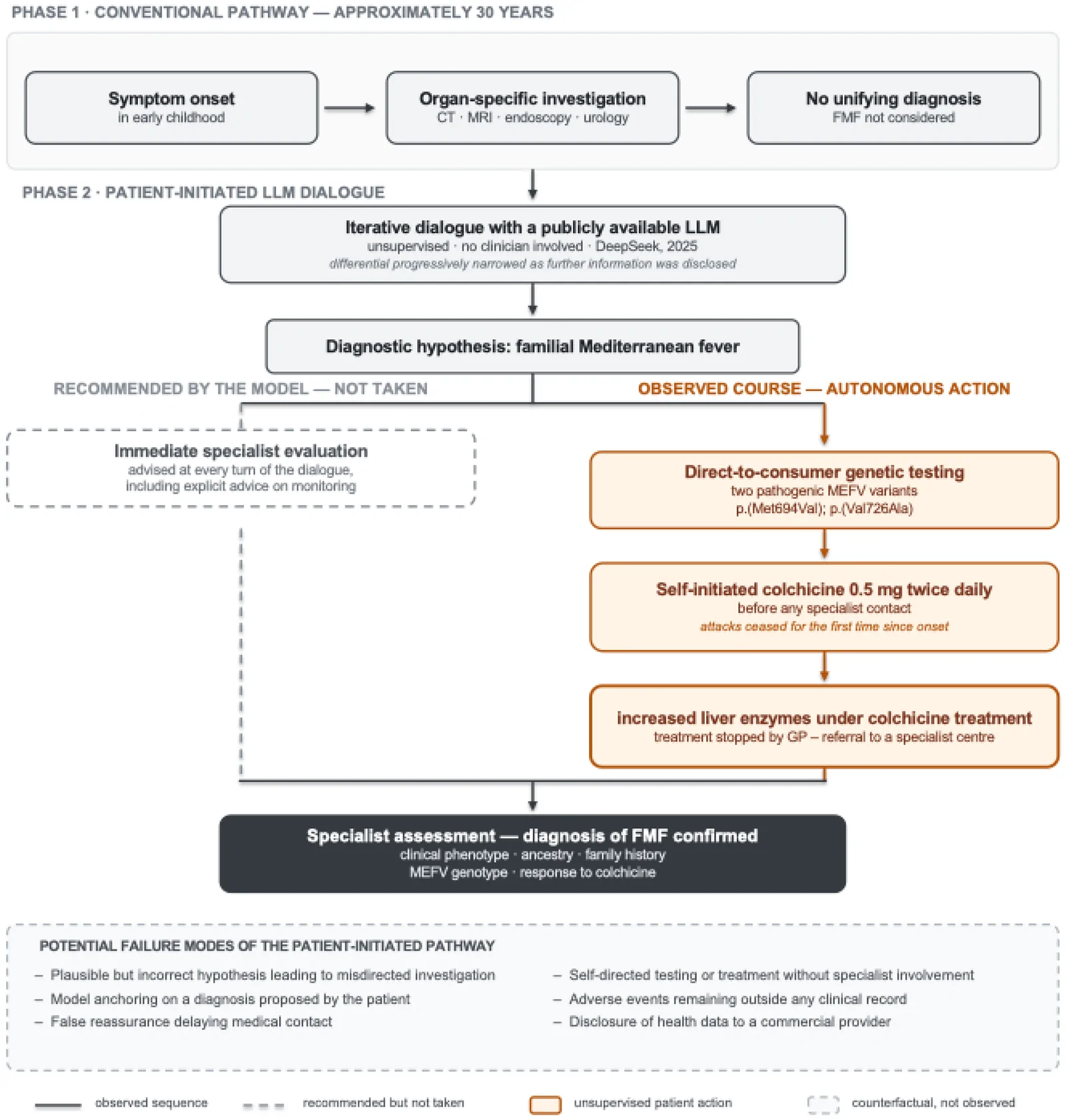
Conceptual comparison of a traditional clinician-led diagnostic pathway and the patient-initiated, LLM-supported pathway illustrated by this case, generated with Claude Opus 5, reasoning settings ‘high’.

## Competing interests

All authors declare they have no competing interests.

## Appendix Supplementary materials

Image, application 1

Image, application 2

## Footnotes

Supplementary material associated with this article can be found in the online version at doi:10.1016/j.ero.2026.100244.

## References

[1] Huo B., Collins G., Chartash D. (2025). Reporting guideline for chatbot health advice studies: the Chatbot Assessment Reporting Tool (CHART) statement. BMJ Med.

[2] Gattorno M., Hofer M., Federici S. (2019). Classification criteria for autoinflammatory recurrent fevers. Ann Rheum Dis..

[3] Hirsch M.C., Ronicke S., Krusche M., Wagner A.D. (2020). Rare diseases 2030: how augmented AI will support diagnosis and treatment of rare diseases in the future. Ann Rheum Dis.

[4] Kremer P., Schiebisch H., Lechner F. (2025). Comparative analysis of large language models and traditional diagnostic decision support systems for rare rheumatic disease identification. EULAR Rheumatol Open.

[5] Hager P., Jungmann F., Holland R. (2024). Evaluation and mitigation of the limitations of large language models in clinical decision-making. Nat Med.

[6] Pillai J., Pillai K. (2023). Accuracy of generative artificial intelligence models in differential diagnoses of familial Mediterranean fever and deficiency of Interleukin-1 receptor antagonist. J Transl Autoimmun.

[7] Yıldız Ç, Küçükali B., Belder N. (2025). Chat GPT vs. rheumatologists: do we still need the clinician?. Kırıkkale Uni Med J.

[8] De Busser B., Roth L., De Loof H. (2025). The role of large language models in self-care: a study and benchmark on medicines and supplement guidance accuracy. Int J Clin Pharm.

[9] Draelos R.L., Afreen S., Blasko B. (2026). Large language models provide unsafe answers to patient-posed medical questions. NPJ Digit Med..

[10] Krusche M., Pankow A., Kubacki T. (2024). AB0031 familial Mediterranean fever in Germany—still a debilitating disease? A multi-centre retrospective analysis. Ann Rheum Dis.

[11] Blank N., Kötter I., Schmalzing M. (2024). Clinical presentation and genetic variants in patients with autoinflammatory diseases: results from the German GARROD registry. Rheumatol Int..

[12] Huo B., Boyle A., Marfo N. (2025). Large language models for chatbot health advice studies: a systematic review. JAMA Netw Open.

