# Supplementary material for: LLM-supported self-diagnosis and self-treatment of familial Mediterranean fever: a case report
Source: EULAR Rheumatol Open. 2026 Sep 29;2(4):100244. doi: 10.1016/j.ero.2026.100244 (PMC13651492; doi:10.1016/j.ero.2026.100244)
Supplement: Supplementary file 2 [file mmc2.docx]

**Supplement 2**

**CHART reporting checklist — Chatbot Assessment Reporting Tool**

U1–U7 denote patient messages and R1–R7 the corresponding model responses. The structure follows the published CHART item numbering (12 items, 39 subitems).

| **Item** | **CHART topic** | **Reported for this case report** |
| --- | --- | --- |
| **TITLE AND ABSTRACT** | | |
| **1a** | **Title** | The title identifies the report as involving a patient-facing large language model (LLM). The design descriptor “case report” is carried in the keywords. |
| **1b** | **Abstract / summary** | The abstract states the model used (DeepSeek), that the interaction was patient-initiated and iterative, the resulting diagnostic hypothesis, the patient’s subsequent autonomous actions and the safety-relevant outcome. |
| **INTRODUCTION** | | |
| **2a** | **Background and rationale** | Patients increasingly consult general-purpose LLMs about unexplained symptoms. Diagnostic delay in familial Mediterranean fever (FMF) outside endemic regions remains substantial. Existing evidence is largely benchmark-based; real-world, patient-initiated use is poorly characterised. |
| **2b** | **Objectives** | To describe a single, unplanned, patient-initiated LLM interaction that preceded recognition of FMF, and the patient’s subsequent autonomous diagnostic and therapeutic actions.  Descriptive case report. No diagnostic accuracy claim is made; no comparator was pre-specified. |
| **METHODS** | | |
| **3a** | **Model identifier** | DeepSeek (DeepSeek-AI), accessed by the patient through the publicly available consumer interface. The retrieved conversation displays the model’s intermediate reasoning before each answer, indicating that the extended-reasoning (“DeepThink”) mode was active. |
| **3b** | **Access, date and setting** | The interaction took place in June 28, 2025, before any specialist contact. Access was by the patient on a personal device, without institutional, clinical or research involvement. The conversation was retrieved by the authors from a share link provided by the patient. |
| **4a** | **Model configuration** | Default consumer configuration. Extended reasoning was active (see 3a). No custom instructions or system prompt were used. No document upload, image upload or file attachment occurred. No external tool use or web citation appears anywhere in the conversation. |
| **4b** | **Model parameters** | Not applicable. The consumer interface was used; temperature, top-p and related decoding parameters were neither user-accessible nor recorded. |
| **4c** | **Fine-tuning or augmentation** | None. A general-purpose, publicly available model was used without retrieval augmentation, domain fine-tuning or any medical plug-in. |
| **5a** | **Prompt engineering** | None. All prompts were composed spontaneously by the patient in lay language, in the first person, without a template, without clinician input and without iterative prompt optimisation. |
| **5b** | **Prompt content and language** | The conversation was conducted entirely in English, which is not the patient’s first language. Prompts were short, unstructured and free of medical terminology. The transcript in Supplement 1 is the original text, reproduced verbatim and without translation; only the patient’s own words appear, and no identifying details were disclosed to the model. |
| **6a** | **Query strategy — structure** | A single continuous conversation comprising seven patient messages and seven model responses. Clinical information was disclosed incrementally and unprompted, in the following order: (U1) childhood-onset migratory abdominal pain with fever, sepsis in 2022, congenital bladder anomaly with corrective surgery in 2024; (U2) multiple sclerosis since 2012, normal contrast-enhanced abdominal CT and MRI; (U3) complete bladder emptying on testing, CRP repeatedly above 100 mg/L during episodes; (U4) normal gastroscopy and colonoscopy, Helicobacter pylori found and eradicated; (U5) multiple sclerosis clinically stable for seven years, treated with fingolimod; (U6) darker urine on days 3–4, complete resolution after four days; (U7) a direct question about the safety of combining colchicine with fingolimod.  Ancestry, family history, arthralgia, myalgia and the erythematous skin lesions described in the case report were never disclosed to the model. The hypothesis was therefore reached from a narrower dataset than that available to the assessing clinicians. |
| **6b** | **Repetition and regeneration** | None. No prompt was repeated, no response was regenerated, and reproducibility was not tested. The exchange was a naturally occurring event, identified as reportable only after the patient presented to specialist care. |
| **6c** | **Memory and context handling** | All seven exchanges took place within one session, so the full history remained in context; the model referred back to earlier disclosures throughout. Whether cross-session memory or personalisation was enabled cannot be determined from the retrieved conversation. |
| **6d** | **Other models or tools** | Not known |
| **7a** | **Performance evaluation** | No formal performance evaluation was undertaken. Model output was not scored against a reference standard and no rating instrument was applied.  The reference diagnosis was established independently by specialist assessment, integrating clinical phenotype, ancestry, family history, MEFV genotype and response to colchicine, in line with the Eurofever/PRINTO classification criteria. |
| **7b** | **Assessors and blinding** | Not applicable. No assessors, no blinding and no independent adjudication; a narrative account described retrospectively by the treating team. |
| **8** | **Sample size** | One patient, one conversation, seven exchanges. No sample size calculation is applicable. The report is descriptive and hypothesis-generating only. |
| **9a** | **Data analysis** | The conversation was read in full and summarised narratively, with attention to the order in which diagnoses were introduced, promoted, demoted and discarded, and to statements bearing on treatment and safety. No coding framework was applied and no inter-rater assessment was performed.  JP read the transcript and MK was verified the transcript. |
| **RESULTS** | | |
| **10a** | **Diagnostic output across the conversation** | R1: a broad differential in seven categories, dominated by urological causes (post-surgical residua, chronic pyelonephritis, perinephric abscess), followed by recurrent peritonitis, abdominal migraine, adhesive obstruction, inflammatory bowel disease, periodic fever syndromes including FMF, and abdominal complex regional pain syndrome.  R2 (after disclosure of multiple sclerosis and normal imaging): neurogenic bladder was named “prime suspect” and urodynamic testing described as “the absolute key next diagnostic step”. FMF was retained but demoted.  R3 (after normal bladder emptying and CRP >100 mg/L): FMF was promoted to leading diagnosis and MEFV testing plus a diagnostic colchicine trial were recommended for the first time.  R4 (after normal endoscopy): inflammatory bowel disease was excluded and FMF described as the “strongest lead”.  R5 (after stable multiple sclerosis on fingolimod): FMF retained as leading diagnosis; multiple sclerosis and its treatment explicitly set aside as unrelated.  R6 (after four-day duration and darker urine): FMF ranked first and TRAPS second; creatine kinase measurement proposed to test for myoglobinuria.  R7: a structured answer on the colchicine–fingolimod combination (see 10b). |
| **10b** | **Treatment and safety output (verbatim)** | Diagnostic confidence, R5: “You have a classic presentation of FMF. A simple colchicine trial could provide life-changing answers and relief.” R4 states: “Your symptom pattern is textbook for conditions like FMF.”  Dosing, R6: “Demand a Diagnostic Trial of Colchicine: Start low dose (e.g., 0.5mg twice daily). If it prevents or drastically shortens your next attack, it’s near-diagnostic for FMF.” This is the dose the patient subsequently self-administered.  Interaction assessment, R7: “There is no known significant direct pharmacokinetic interaction between colchicine and fingolimod (Gilenya).” The model additionally advised beginning at 0.5 mg once daily and up-titrating only if tolerated — a more cautious schedule than the one the patient adopted.  Monitoring, R7: baseline complete blood count, comprehensive metabolic panel including creatinine and liver function tests, creatine kinase and urinalysis were specified before starting colchicine, with repeat testing “within 1–3 months of starting, then periodically”. None of these were obtained.  Safety qualification, R7: “Do not start colchicine without your rheumatologist and neurologist explicitly agreeing on the plan and monitoring.” Specialist referral was recommended in every one of the seven responses. |
| **10c** | **Errors, omissions and uncertainty** | Three shortcomings were identified. First, R2 constituted a regression: after disclosure of multiple sclerosis the model promoted neurogenic bladder to prime suspect and urged urodynamic testing, an investigation the patient had already undergone; the correct trajectory resumed only after the patient volunteered the corrective information.  Second, the reasoning text preceding R4 asserts that the patient’s “Mediterranean heritage [was] mentioned in previous conversation”. Ancestry was never disclosed at any point. This is a fabricated premise supporting a conclusion that was nevertheless correct.  Third, investigations were recommended that a specialist would be unlikely to prioritise once the phenotype had emerged, and several were repeated after FMF had become the leading diagnosis: PET/CT, indium-111 labelled leucocyte scintigraphy, capsule endoscopy, abdominal angiography, ANCA and antinuclear antibody testing, pelvic floor physiotherapy and pain management referral.  No factually incorrect clinical statement was identified. |
| **DISCUSSION** | | |
| **11a** | **Interpretation** | The model revised its differential in response to sequentially disclosed information and produced a hypothesis that proved correct, from a dataset narrower than that available to the assessing clinicians. Its safety instructions were specific: it named the required baseline investigations, proposed a more cautious starting dose than the patient adopted, and conditioned treatment on specialist agreement in every response.  The dissociation between adequate model output and inadequate patient adherence is the central observation of this report. |
| **11b** | **Limitations** | Single case with a favourable outcome; selection and reporting bias are unquantifiable. The conversation reflects only what the patient chose to disclose. Attribution of the patient’s subsequent actions to specific model outputs rests on temporal sequence and retrospective self-report. The model checkpoint cannot be reconstructed and the interaction is therefore not reproducible. |
| **11c** | **Generalisability** | None is claimed. The report describes one trajectory in which the hypothesis happened to be correct and specialist care was ultimately reached. Neither the diagnostic accuracy nor the safety of patient-facing LLMs can be inferred from a single observation. |
| **OPEN SCIENCE** | | |
| **12a** | **Disclosures** | All other authors report no conflicts of interest |
| **12b** | **Funding** | No specific grant was received from funding agencies in the public, commercial or not-for-profit sectors. |
| **12c** | **Ethics and consent** | Formal ethics committee approval was not required for a single-patient case report under institutional and national regulations. Written informed consent was obtained for publication of the clinical details and of the transcript.  No anonymisation of the transcript was necessary: the patient disclosed no name, date of birth, place of residence, institution or other identifier to the model. The conversation nevertheless constitutes transmission of special-category health data to a commercial provider outside any clinical relationship, which occurred before the treating team had any involvement. |
| **12d** | **Protocol and registration** | None. The interaction was not planned, prospectively designed or registered. |
| **12e** | **Data availability** | The complete conversation, comprising all seven exchanges together with the model’s displayed reasoning, is reproduced unabridged in Supplement 1. It was retrieved from a share link supplied by the patient; because such links may expire or be withdrawn, the archived text in the supplement is the definitive record. |
| **DECLARATION OF AI USE IN MANUSCRIPT PREPARATION** | | |
| **—** | **Authoring tools** | ChatGPT 5.5 was used for language editing and structural suggestions during manuscript preparation. All output was reviewed and edited by the authors, who take full responsibility for the final content. |

**Note on reproducibility**

Consumer LLM interfaces are updated continuously and do not expose model version, decoding parameters or system prompts to the user. An interaction of this kind is therefore not reproducible in the sense intended by CHART. We report what could be established and identify explicitly what could not, rather than omitting the items.
